# Chronic Lithium Treatment Affects Anxious Behaviors and theExpression of Serotonergic Genes in Midbrain Raphe Nuclei of Defeated Male Mice

**DOI:** 10.3390/biomedicines9101293

**Published:** 2021-09-22

**Authors:** Dmitry A. Smagin, Irina L. Kovalenko, Anna G. Galyamina, Irina V. Belozertseva, Nikolay V. Tamkovich, Konstantin O. Baranov, Natalia N. Kudryavtseva

**Affiliations:** 1 FRC Institute of Cytology and Genetics, Siberian Branch of Russian Academy of Sciences, 630090 Novosibirsk, Russia; smagin@bionet.nsc.ru (D.A.S.); koir@bionet.nsc.ru (I.L.K.); galyamina@bionet.nsc.ru (A.G.G.); 2 Valdman Institute of Pharmacology, First Pavlov State Medical University of St. Petersburg, 197022 St. Petersburg, Russia; beloiz@spmu.rssi.ru; 3 Biolabmix, 630090 Novosibirsk, Russia; nv_tamk@niboch.nsc.ru; 4 Institute of Molecular and Cellular Biology, Siberian Branch of Russian Academy of Sciences, 630090 Novosibirsk, Russia; baranov@mcb.nsc.ru; 5 Pavlov Institute of Physiology, Russian Academy of Sciences, 188680 St. Petersburg, Russia; 6 Head of Neuropathology Modeling Laboratory, Institute of Cytology and Genetics SB RAS, pr. Ac. Lavrentjev, 10, 630090 Novosibirsk, Russia

**Keywords:** chronic social defeat stress, anxiety, depression, lithium chloride, mice

## Abstract

There is experimental evidence that chronic social defeat stress is accompanied by the development of an anxiety, development of a depression-like state, and downregulation of serotonergic genes in midbrain raphe nuclei of male mice. Our study was aimed at investigating the effects of chronic lithium chloride (LiCl) administration on anxiety behavior and the expression of serotonergic genes in midbrain raphe nuclei of the affected mice. A pronounced anxiety-like state in male mice was induced by chronic social defeat stress in daily agonistic interactions. After 6 days of this stress, defeated mice were chronically treated with saline or LiCl (100 mg/kg, i.p., 2 weeks) during the continuing agonistic interactions. Anxiety was assessed by behavioral tests. RT-PCR was used to determine *Tph2, Htr1a, Htr5b,* and *Slc6a4* mRNA expression. The results revealed anxiolytic-like effects of LiCl on social communication in the partition test and anxiogenic-like effects in both elevated plus-maze and social interaction tests. Chronic LiCl treatment upregulated serotonergic genes in midbrain raphe nuclei. Thus, LiCl effects depend on the treatment mode, psycho-emotional state of the animal, and experimental context (tests). It is assumed that increased expression of serotonergic genes is accompanied by serotonergic system activation and, as a side effect, by higher anxiety.

## 1. Introduction

Lithium salts are widely used in psychiatric practice in monotherapy regimens as mood stabilizers [1,2,3,4], in the supportive therapy of psycho-emotional disorders [5], and in the prevention of suicidal behaviors in patients [6,7,8]. Lithium is also used at the onset of the depressive phase in bipolar disorders and for the prevention of mood disorders [3,9,10,11,12] or relapses in schizophrenia with aggressive or suicidal behavior, convulsions, and other health problems [13,14,15,16]. Nevertheless, according to these studies, in clinical practice, patients with bipolar disorder demonstrate two types of lithium responsiveness: they are either good or poor responders. Lithium as monotherapy or in combination with other drugs is effective in 60% of chronically treated patients, but the treatment response remains heterogeneous and a large number of patients require a change in treatment after several weeks or months.

Lithium is obviously a multitarget drug and, as a consequence, with multiple mechanisms of action [17,18], and this property complicates the elucidation of its mechanism of action in a given context. The latest findings revealed numerous genes associated with a lithium response in bipolar disorder [18,19,20,21,22]. Therefore, there is a substantial need for tools that can guide clinicians in selecting a correct treatment strategy and that can aid in understanding individual differences in the response to lithium in clinical practice. We agree that important questions regarding the mechanism of lithium action on anxiety and depression remain open [17,18], despite much practical application.

In our previous study, a lithium-based enterosorbent called “Noolit” had obvious anxiolytic and antidepressant effects in adult defeated male mice [23]. In the present study, we provide experimental data on the impact of chronic treatment with lithium chloride (LiCl) on the anxiety-like state caused by chronic social defeat stress that leads to a mixed anxiety/depression-like state in male mice [24,25,26]. Obvious similarities in symptoms (general behavioral deficits, helplessness, anxiety, and decreased communication), etiology, sensitivity to antidepressants and anxiolytics (imipramine, fluoxetine, and diazepam), and serotonergic changes in the brain were demonstrated here between mice with clinical manifestations and patients. Our current results will be compared with the effects of the chronic lithium treatment seen in similar previous experiments on male mice with repeated experiences of aggression in daily agonistic interactions, which are accompanied by the development of a whole range of changes in behavior and a psycho-emotional state. Chronically aggressive male mice are known to demonstrate an increased number of stereotypical behaviors including enhanced anxiety, hyperactivity, and strong aggressive motivation, which, along with other signs, indicate the development of psychosis-like behavior [27,28].

There is evidence that therapeutic action of lithium is due to the effects on serotonergic neurotransmission [29,30]. Studies on humans have demonstrated that the effects of lithium on the serotonergic system depend on tryptophan hydroxylase variants [11] and that lithium may act through 5-HT_1B_ receptors, as shown in animal models [31]. Regular administration of lithium increases the density of the serotonin uptake site in cortical regions, suggesting an increase in the number of serotonin transporters in the brain regions containing nerve terminals of serotonergic neurons [32].

In the present study, we took into account many of the recently obtained data on the existence of lithium-sensitive genes that may be involved in the development of affective and neurodegenerative disorders [19,20,21,22,33,34]. Our previous experiments [35] revealed the downregulation of serotonergic gene expression in midbrain raphe nuclei of male mice with defeated experience in daily agonistic interactions, which induced the development of anxiety and depression-like states. The aim of the current work was to study the effect of chronic administration of LiCl on anxiety-like behaviors and expressions of serotonergic genes in this brain region. Considering lithium is used at the initial stage of the depressive phase of bipolar disorder and for the prevention of mood diseases in patients [10,11,12], in our experiment, we administered LiCl during the period of repeated agonistic interactions, which are accompanied by the development of an anxiety-like state from the first day of experiencing defeats.

## 2. Methods

### 2.1. Animals

Adult C57BL/6 male mice were obtained from the Animal Breeding Facility, a branch of the Institute of Bioorganic Chemistry, RAS (Pushchino, Moscow region, Russia). The animals were housed under standard conditions (12:12 h light/dark cycle, lights on at 8.00 a.m.; food (pellets) and water were available ad libitum). The mice were weaned at 1 month of age and housed in groups of 8–10 in plastic cages (36 × 23 × 12 cm). All procedures were carried out in compliance with the international regulations for animal experiments (Directive 2010/63/EU of the European Parliament and of the Council on the Protection of Animals Used for Scientific Purposes). The protocol for the study was approved by Scientific Council No. 9 of the Institute of Cytology and Genetics, SB RAS, of 24 March 2010, N 613 (Novosibirsk).

### 2.2. Chronic Social Defeat Stress and LiCl Treatment

Prolonged exposure to chronic social defeat stress that is accompanied by a strong anxiety-like state in male mice was induced in accordance with the sensory contact model [24,28]. Pairs of weight-matched animals were each placed in a cage (28 × 14 × 10 cm) bisected by a perforated transparent partition, allowing the animals to see, hear, and smell each other but preventing physical contact. The animals were left undisturbed for 3 days to adapt to the new housing conditions and sensory contact before they were subjected to an encounter. Then, every afternoon (2:00–5:00 p.m., local time), the cage lid was replaced by a transparent one, and 5 min later (the period necessary to stimulate mouse activity), the partition was removed for 10 min to encourage agonistic interactions. The superiority of one of the mice was firmly established within two or three encounters with the same opponent. The superior aggressive mouse was attacking, biting, and chasing the other mouse, who was displaying only defensive behavior (upright postures, sideways postures, freezing or withdrawal, and lying on the back). As a rule, the agonistic interactions between the two males were discontinued by lowering the partition if the aggression lasted for 3 min or in some cases even less. Each defeated mouse (loser) was exposed to the same aggressive mouse (winner) for 3 days. Afterwards, each loser was placed once a day after the fight in an unfamiliar cage with an unfamiliar aggressive mouse behind the partition. Each winning mouse remained in its original cage. This procedure was performed once a day and yielded equal numbers of winners and losers.

**Figure 1:**
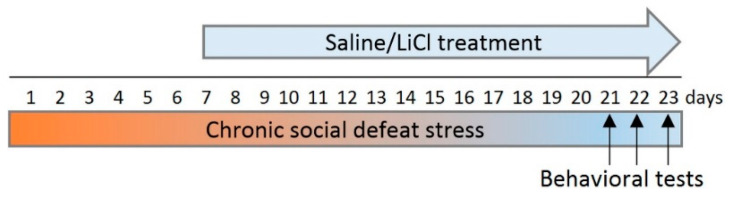
An outline of the experiment: chronic LiCl and/or saline treatment of defeated mice in preventive mode starting from the 7th day of the agonistic interactions in order to study the possible protective effect of LiCl on the development of behavioral pathology. Behavioral tests: day 21, the partition test; day 22, the elevated plus-maze test; and day 23, the social interaction test.

To investigate the effect of LiCl on anxiety-like behavior, we employed an experimental approach that is used for the screening of psychotropic drugs in settings mimicking clinical conditions [36,37]. This pharmacological approach makes it possible to study protective properties of drugs used in the preventive mode (Figure 1) and their therapeutic properties in animals having a behavioral pathology.

LiCl was administered during the period of the repeated agonistic interactions and we expected to see its protective effects. For this purpose, after 6 days of the agonistic interactions accompanied by chronic social defeat stress, the defeated mice were treated with either saline or LiCl (Merck, Germany) at a dose of 100 mg/kg intraperitoneally once a day in the morning (9.00–10.00 a.m., local time). Three groups of animals were used in the behavioral experiment (Figure 1): (i) controls, i.e., mice without consecutive experiences of agonistic interactions; (ii) defeated males chronically treated with saline (Sal-treated losers); and (iii) defeated males chronically treated with LiCl (LiCl-treated losers). After 2 weeks of the LiCl or saline injections against the background of agonistic interactions, the behavior of the animals was evaluated in behavioral tests (one test per day), which were used for quantifying the anxiety-like state in different experimental conditions. 

### 2.3. Behavioral Tests

#### 2.3.1. The Partition Test

This test can be utilized for the estimation of a mouse behavioral reaction to a conspecific behind the transparent perforated partition dividing the experimental cage into equal parts [38]. The number of approaches to the partition and the total time spent near it (e.g., moving near the partition, smelling and touching it with the nose or with one or two paws, and sticking the nose into the holes) were scored during 5 min as indices of reacting to the partner. The duration of a sideways position or “turning away” near the partition was not included in the total time of the test. The experimental procedure was as follows: the pair of mice resided together in a cage with a partition. On the testing day, the lid of the cage was replaced by a transparent one; 5 min later (period of activation), behavioral responses of the losers and controls toward the unfamiliar partner were recorded for 5 min. This test is used in the research on communicativeness (sociability) and anxiety: it has been shown that a decrease of partition parameters correlates with indices of anxiety-like behavior, estimated in the elevated plus-maze test [38].

#### 2.3.2. The Elevated Plus-Maze Test 

The elevated plus-maze consisted of two open arms (25 × 5 cm) and two closed arms (25 × 5 × 15 cm), and was placed in a dimly lit room. The two arms of each type were opposite to each other and extended from a central platform (5 × 5 cm). The maze was elevated by 50 cm above the room floor [39].

The cover of the experimental cage with a mouse was replaced by a transparent lid in the same room 5 min before placement in the plus-maze. The mouse was placed on the central platform with the nose to the closed arm. The following measures were recorded for 5 min: (1) total entries; (2) open arm entries (four paws in an open arm), closed arm entries (four paws in a closed arm), and central platform entries; (3) time spent in open arms, closed arms, and the central platform (center); (4) the number of passages from one closed arm to another; (5) the number of head dips (looking down on the floor below the plus-maze); and (6) the number of peeping-out instances when the mouse was in closed arms. Indices 2 and 3 are considered measures of the level of anxiety, indices 1 and 4 are related to locomotor activity, and indices 5 and 6 quantify the risk assessment behavior. The time spent in closed arms and open arms, and on the central platform (center) was calculated as percentages of the total testing time. The elevated plus-maze was thoroughly cleaned between the sessions.

#### 2.3.3. Exploratory Activity and Social Interaction Tests 

An open field (36 × 23 cm) was used with a perforated container (an inverted pencil holder made of metal wire, bottom diameter: 10.5 cm) in one of the cage corners. Each mouse was placed individually in the corner opposite to the pencil holder for 5 min. This test allows to estimate the exploratory behavior of mice under novel conditions with an unfamiliar object, namely the pencil holder [40]. This test is thought to cause pronounced stress. Then, an unfamiliar male from the housed group was placed under the pencil holder for 5 min to study the reaction of the male mouse to the conspecific animal in a familiar situation (social interaction test).

The EthoVision XT software (version 11.0; Noldus Information Technology, Wageningen, The Netherlands) automatically registered the tracking score (distance) during the testing time with differentiation of the place near the pencil holder (5 cm around it) in the cage as well as the total time spent in the corner opposite to the pencil holder.

Manual registration with Observer XT (version 7.0; Noldus Information Technology, the Netherlands) of the following behavioral indicators of communicativeness was carried out: the number and/or duration of (1) rearings (exploratory activity); (2) groomings (self-oriented behavior: licking of the fur on the flanks or abdomen and washing over the head from an ear to snout); and (3) approaches to the pencil holder and total time (s) spent near it (moving near the pencil holder, smelling and touching it with the nose or with one or more paws). The duration of a sideways position or “turning away” near the pencil holder was not included in the total time. After each test, the open field and pencil holder were thoroughly washed and dried off with napkins.

Preliminary analysis of LiCl-treated losers’ behavior in the exploratory activity test clearly stratified the animals into two groups: LiCl-sensitive - LiCl^+^-treated and LiCl-less-sensitive - LiCl^−^-treated losers in relation to the chronic LiCl treatment. As a behavioral parameter for the division into groups, we employed avoidance of a novel object (pencil holder): LiCl^+^-treated losers did not approach the pencil holder at all, sat in the corner only, and did not explore the cage. Among the controls as well as the Sal-treated and LiCl^−^-treated losers, we observed a natural exploratory activity under the novel conditions.

### 2.4. Real-Time Polymerase Chain Reaction (RT-PCR)

To understand the possible effect of LiCl on the behavior of the experimental animals, we conducted an additional experiment that, we hoped, would explain the effect of LiCl (150 mg/kg) on the expression of serotonergic genes in midbrain raphe nuclei, which contain bodies of serotonergic neurons. The expression of the following genes was analyzed: *Tph2*, encoding tryptophan hydroxylase, which is a rate-limiting enzyme of serotonin synthesis; *Slc6a4*, encoding a serotonin transporter; and genes *Htr1a* and *Htr5b*, encoding serotonin receptors. To measure mRNA levels of serotonergic genes in midbrain raphe nuclei, we studied the 21-day losers after chronic LiCl or saline injections at 24 h after the last agonistic interaction, alongside the controls.

The determination of serotonin-related gene expression in midbrain raphe nuclei by RT-PCR was done at the Biolabmix Company (https://biolabmix.ru (accessed on 17 March 2020), Novosibirsk, Russia). The measurement data were provided by the Bio-Rad Amplifier software (Berkeley, CA, USA). RNA was isolated using the TRIzol reagent (Invitrogen, Waltham, MA, USA). To remove DNA impurities, the obtained RNA samples were treated with DNase I (Fermentas) for 1 h at 37 °C according to the manufacturer’s protocol and the enzyme was inhibited by adding EDTA and heating at 65 °C for 10 min. The quality of the isolated RNA was checked spectrophotometrically and its integrity by electrophoretic mobility in a 2% agarose gel. The absence of genomic DNA was confirmed for each sample by PCR. The cDNA was prepared from each RNA sample in two parallel reactions in a volume of 20 μL using the M-MuLV–RH First Strand cDNA Synthesis Kit (Biolabmix, Novosibirsk, Russia). RNA (1 μg), 100 U of MuMLV reverse transcriptase (murine leukemia virus reverse transcriptase), and 0.3 μM random hexoprimer were used in the reaction. The reaction was carried out according to the manufacturer’s protocol. The level of RNA expression in the samples was assessed by RT-PCR with fluorescent probes. For this purpose, primers specific to the four functional genes under study (*Tph2, Slc6a4, Htr1a,* and *Htr5b*) were used (Table S1).

PCR was conducted in a 25 μL reaction solution containing 2× BioMaster HS-qPCR (2×) reaction mixture (Biolabmix, Novosibirsk, Russia), an aliquot of the reaction mixture after reverse transcription, 300 nM forward and reverse primers, and 200 nM fluorescent probe. The amplification was performed on the Real-Time CFX96 Touch (Bio-Rad, Berkeley, CA, USA) according to the following program: 1st cycle consisted of 5 min at 95 °C; 45 cycles consisted of 20 s at 95 °C and 60 s at 60 °C". Each sample was amplified in triplicates. 

In animals of all experimental groups after quick decapitation, the midbrain raphe nuclei area was dissected according to the map presented in the Allen Mouse Brain Atlas (http://mouse.brain-map.org/static/atlas (accessed on 24 April 2005). Dissection of the brain region was made by the same experimenter. The brain regions were removed and chilled rapidly on ice. All biological samples were encrypted, rapidly frozen in liquid nitrogen, and stored at −70 °C until use. 

### 2.5. Statistical Analysis

The analysis of the behavioral data was performed by either one-way ANOVA for parametric variables or by the Kruskal–Wallis test. The Kruskal–Wallis test was conducted with “group” as a factor (controls, Sal-treated losers, LiCl^−^-treated losers, and LiCl^+^-treated losers) for the social interaction test and with “group” as a factor (controls, Sal-treated losers, and LiCl-treated losers) for both the partition and elevated plus-maze tests, which were followed by either Tukey’s multiple-comparison post hoc test for parametric variables or by Dunn’s multiple-comparison post hoc test if the parametric criteria were not met. To display the variance of the values, the data are presented as a box-whisker plot showing means (*plus sign*), medians (*solid lines*), and 25%/75% quartiles, with whiskers indicating 10th and 90th percentiles. All statistical analyses were performed using the XLStat software (Addinsoft, www.xlstat.com (accessed on 31 March 2016)). For the parameter “number of entries” (four paws in open arms) in the elevated plus-maze test, the chi-square test was used.

## 3. Results

### 3.1. Experiment 1. Effects of Chronic LiCl Treatment on Anxious Behavior of Defeated Mice

#### 3.1.1. Effects of Chronic LiCl Treatment on the Behavior of Defeated Mice in the Partition Test 

**Figure 2:**
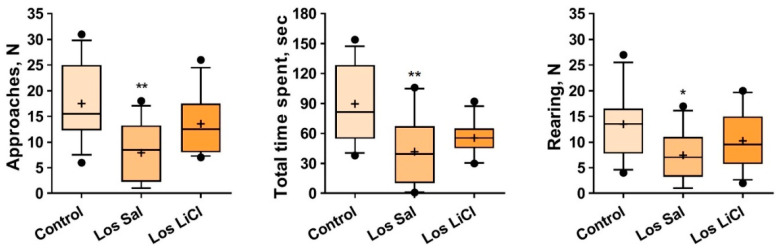
Effects of chronic LiCl treatment on the behavior of defeated mice in the partition test. Note: Los Sal-Sal-treated losers; and Los LiCl-LiCl-treated losers. Data are presented as means (*plus sign*), medians (solid lines), and 25%/75% quartiles in the box-whisker plot, with whiskers indicating the 10th and 90th percentiles. * *p* < 0.05 and ** *p* < 0.01 vs. controls; *n* = 12 for each group.

One-way ANOVA revealed an influence of the “group” factor on the numbers of approaches (F(2,33) = 6.619, *p* = 0.0038) and rearings (F(2,33) = 3.293, *p* = 0.0496). The Kruskal–Wallis test uncovered an impact of the “group” factor on the total time spent near the partition (H = 9.816, *p* = 0.0074). According to either Tukey or Dunn’s multiple-comparison post hoc test, significant differences between the Sal-treated losers and controls were found in the number of approaches (*p* = 0.0027), in the total time spent near the partition (*p* = 0.0053), and in the number of rearings (*p* = 0.0390) (Figure 2).

Thus, in comparison with the control, Sal-treated losers demonstrated lower behavioral activity (communication and sociability), as evidenced by the number of approaches to and total time spent near the partition as a reaction to the partner in the neighboring compartment as well as by the decreased exploratory activity, estimated as the number of rearings. After the LiCl treatment, these parameters did not differ significantly from the control. We can assume that LiCl had slight anxiolytic effects on the losers.

#### 3.1.2. Effects of Chronic LiCl Treatment on the Behavior of Defeated Mice in the Elevated Plus-Maze Test 

**Figure 3:**
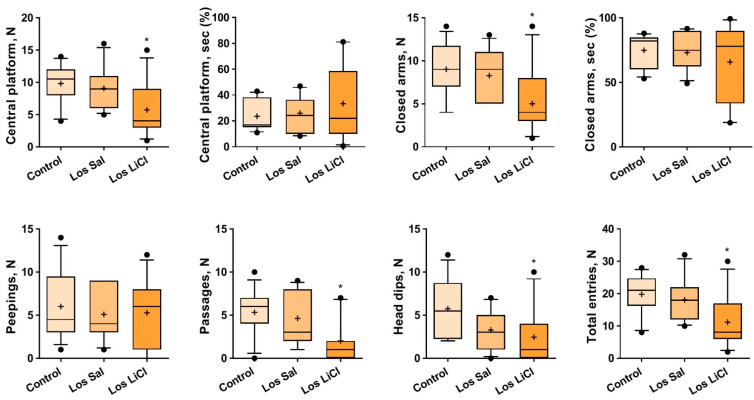
Effects of chronic LiCl treatment on the behavior of defeated mice in the elevated plus-maze test. Note: Los Sal-Sal-treated losers; and Los LiCl-LiCl-treated losers. * *p* < 0.05 vs. controls, Tukey’s multiple-comparison post hoc test (*n* = 12 for each group). The presented values are means (plus sign), medians (solid lines), and 25%/75% quartiles in the box-whisker plot, with whiskers indicating the 10th and 90th percentiles.

One-way ANOVA revealed a significant influence of “group” (controls, Sal-treated losers, and LiCl-treated losers) on the numbers of central platform entries (F(2,31) = 4.130, *p* = 0.0257), closed arm entries (F(2,31) = 4.661, *p* = 0.0170), passages (F(2,31) = 4.717, *p* = 0.0163), head dips (F(2,31) = 3.590, *p* = 0.0396), and total entries (F(2,31) = 4.552, *p* = 0.0185). Tukey’s multiple-comparison *post hoc* test detected differences between the LiCl-treated and control mice in the numbers of central platform entries (*p* = 0.0277), closed arm entries (*p* = 0.0185), passages (*p* = 0.0166), head dips (*p* = 0.0403), and total entries (*p* = 0.0192) (Figure 3). The chi-square test did not reveal differences between experimental groups in the parameter “number of entries” (four paws in open arms). 

Therefore, LiCl caused a decrease in all parameters of locomotor activity; this effect can be easily explained by the general behavioral deficit that develops within a depression-like state in mice [24,25,26]. This effect of LiCl may also be considered pro-depressive in this experimental context.

Previously, it has been repeatedly reported that well-pronounced anxiety-like behavior develops in the losers after 20-day social defeat stress, as estimated by the elevated plus-maze test [26,36]. During the chronic saline treatment in our study, these negative changes were less pronounced. It can be cautiously concluded that saline has a protective effect when administered as chronic injections. By contrast, the general decrease in locomotor activity during our chronic treatment with LiCl can be regarded as an anxiogenic effect.

#### 3.1.3. Effects of Chronic LiCl Treatment on the Exploratory Activity of Defeated Mice with Different Sensitivities to LiCl in the Novel Situation toward the Unfamiliar Object (Empty Pencil Holder)

**Figure 4:**
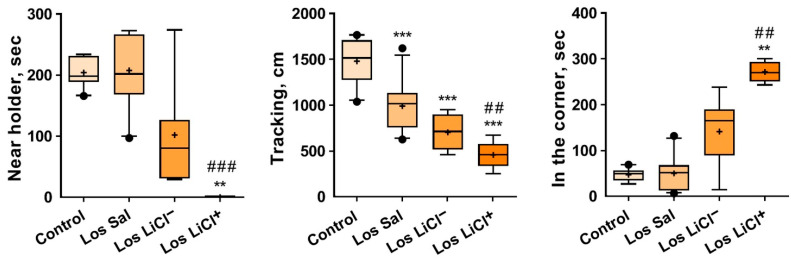
Effects of LiCl on the exploratory behavior of defeated mice in the novel environment and towards the novel object (pencil holder). Note: Control (*n* = 9); Los Sal-Sal-treated losers (*n* = 11); Los LiCl-LiCl-treated losers (*n* = 7); Los LiCl^+^-LiCl^+^-treated losers (*n* = 5). The values are means (plus sign), medians (solid lines), and 25%/75% quartiles in the box-whisker plot, with whiskers indicating the 10th and 90th percentiles. ** *p* < 0.01, *** *p* < 0.01 vs. controls; ^##^ *p* < 0.01, and ^###^ *p* < 0.001 vs. Sal-treated losers (Tukey’s multiple-comparison post hoc test).

According to the level of pencil holder avoidance (see the description in Materials and Methods), we subdivided the losers after LiCl injections into two subgroups: LiCl^+^-treated and LiCl^−^-treated losers. One-way ANOVA revealed an impact of the factor “group” (controls or Sal-treated, LiCl^−^-treated, or LiCl^+^-treated losers) on the total tracking score (cm; F(3,29) = 26.50, *p* < 0.0001). Tukey’s multiple-comparison test showed significant differences between the Controls and Sal-treated (*p* = 0.0003), LiCl^−^-treated (*p* < 0.0001), or LiCl^+^-treated losers (*p* < 0.0001), as well as between LiCl^+^-treated and Sal-treated losers (*p* = 0.0013). The Kruskal–Wallis test detected an influence of the “group” factor on the time spent in the corner (H = 18.03, *p* = 0.0004) and near the pencil holder (H = 17.56, *p* = 0.0005). Dunn’s multiple-comparison test detected differences in the following parameters: the time (s) spent in the corner of Controls vs. LiCl^+^-treated losers (*p* = 0.0027); Sal-treated losers vs. LiCl^+^-treated losers (*p* = 0.0012)] and time (s) spent near the pencil holder (Controls vs. LiCl^+^ treated losers (*p* = 0.0040)); and Sal-treated losers vs. LiCl^+^-treated losers (*p* = 0.0010)] (Figure 4).

The LiCl^+^-treated losers were more sensitive to LiCl and exhibited lower exploratory activity, as estimated by the total tracking time. They spent most of the time in the corner and were never near the pencil holder. Together with the behavior in the plus-maze and partition tests, these data are suggestive of decreased exploratory activity and an enhanced anxiety-like state after chronic LiCl treatment.

#### 3.1.4. The Impact of Chronic LiCl Treatment on the Reaction of Mice to an Unfamiliar Partner in the Social Interaction Test 

**Figure 5:**
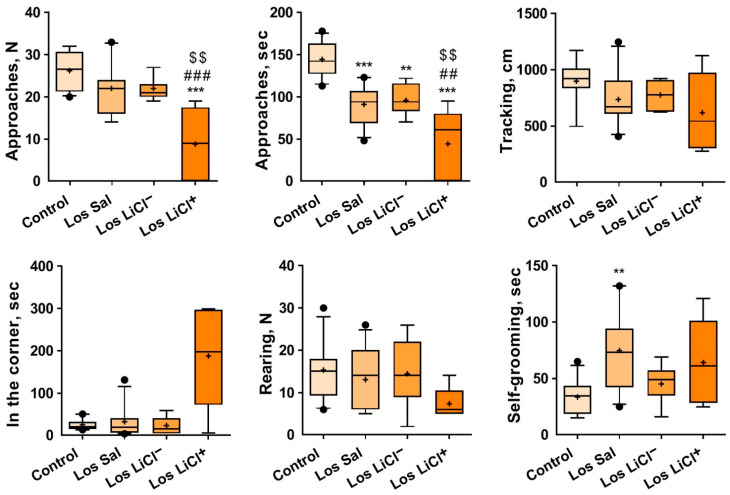
Effects of LiCl on the behavior of defeated mice in the social interaction test as a reaction to the partner under the pencil holder. Note: Control (*n* = 9); Los Sal-Sal-treated losers (*n* = 11); Los LiCl^−^-LiCl^−^-treated losers (*n* = 7); Los LiCl^+^-LiCl^+^-treated losers (*n* = 5). The data are shown as means (plus sign), medians (solid lines), and 25%/75% quartiles in the box-whisker plot, with whiskers indicating the 10th and 90th percentiles. ** *p* < 0.01, *** *p* < 0.001 vs. controls; ^##^ *p* < 0.01, and ^###^ *p* < 0.001 vs. Sal-treated losers; ^$$^ *p* < 0.01 vs. LiCl^−^-treated losers.

One-way ANOVA indicated a significant influence of the factor “group” (controls or Sal-treated, LiCl^−^-treated, or LiCl^+^-treated losers) on the number of approaches to a partner (F(3,31) = 11.35, *p* < 0.0001) and on the total time spent near the pencil holder (approaches; F(3,31) = 20.98, *p* < 0.0001). The Kruskal–Wallis test uncovered an impact of the “group” factor on the duration of self-grooming (H = 9.816, *p* = 0.0212). Tukey’s multiple-comparison test was applied to the following behavioral parameters (Figure 5): approaches *(n* (controls vs. LiCl^+^-treated losers [*p* < 0.0001]; Sal-treated losers vs. LiCl^+^-treated losers [*p* = 0.0008]; and LiCl^−^-treated vs. LiCl^+^-treated losers [*p* = 0.0020])) and approaches (sec (controls vs. Sal-treated losers [*p* < 0.0001], LiCl^−^-treated losers [*p* = 0.0017], or LiCl^+^-treated losers [*p* < 0.0001]; Sal-treated losers vs. LiCl^+^-treated losers [*p* = 0.0083]; and LiCl^−^-treated losers vs. LiCl^+^-treated losers [*p* = 0.0073])). Dunn’s multiple-comparison test was applied to the duration (sec) of self-grooming (controls vs. Sal-treated losers (*p* < 0.0133)) (Figure 5).

We believe that this test measures the level of communicativeness towards an unfamiliar partner under the conditions that have already become familiar during the 5 min before the introduction of the partner. During the preceding 5 min, the mouse realized that it was not in danger and soon began to examine the cage.

Chronic LiCl treatment had a strong anxiogenic effect on LiCl^+^-treated losers in comparison with the controls and Sal-treated losers, as evidenced by the decreased number of approaches to and total time spent near the pencil holder containing a partner (approaches, sec). Their time spent in corners was significantly longer in comparison with all other groups. The anxiogenic effects were significantly less different between the LiCl^−^ -treated losers and control mice.

### 3.2. Experiment 2. Effects of the Chronic LiCl Treatment on the Expression of Serotonergic Genes in the Midbrain Raphe Nuclei of Defeated Mice 

**Figure 6:**
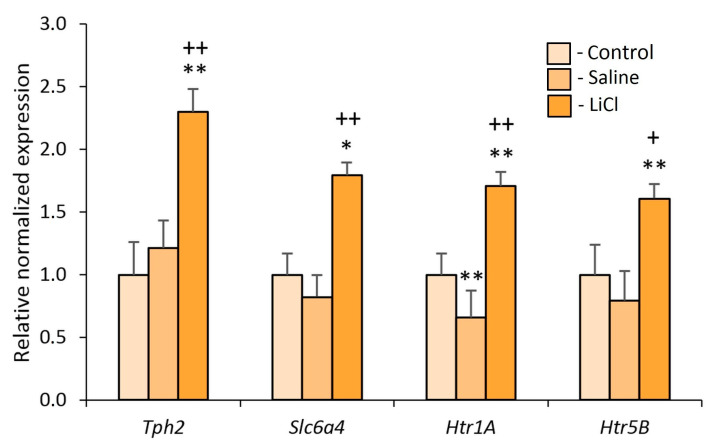
The influence of chronic LiCl treatment on serotonergic gene expression in midbrain raphe nuclei of defeated mice. The measurement data were processed in the Bio-Rad Amplifier software (USA). Bars: the control (*n* = 9); Saline - Sal-treated losers (*n* = 7); LiCl - LiCl-treated losers (*n* = 9). * *p* < 0.05 and ** *p* < 0.01 vs. controls, and ^+^ *p* < 0.05 and ^++^ *p* < 0.01 vs. Sal-treated losers according to Student’s *t*-test. The data are presented as mean ± SEM.

In comparison with the controls and Sal-treated losers, chronic LiCl treatment induced overexpression of the *Tph2* gene (*p* < 0.01 for both), *Slc6a4* gene (*p* < 0.05 and *p* < 0.01), *Htr1a* gene (*p* < 0.01 for both), and *Htr5b* gene (*p* < 0.01 and *p* < 0.05) in the midbrain raphe nuclei of defeated mice (Figure 6).

Some discrepancy between the data obtained earlier [35] and our current results can be explained by slight experimental differences. In the earlier study, we did not chronically administer saline to the losers but we did in the present experiment. There is also the possibility that saline may have a protective impact, similar to those in the elevated plus-maze test, with chronic injections attenuating the adverse effects of chronic social defeat stress.

## 4. Discussion

An important finding here is the varied sensitivity to a drug in mice of the same group, in particular regarding LiCl in this work. When evaluating the behavior of aggressive [41] and defeated male mice (this study) in the social interaction test, we found that mice of one group can be subdivided into subgroups according to differential sensitivity to LiCl: only 40% of chronically aggressive and defeated males were sensitive to chronic lithium treatment. Naturally, a question arises: why did the group of inbred mice split into groups of sensitive and less sensitive to the drug under obviously identical experimental conditions? One plausible reason, as supposed in References [42,43], concerns the differences in prenatal and early postnatal development, which have been overlooked in the standardized experimental setting. In our opinion, however, a more likely assumption is that baseline psycho-emotional states were different among the experimental animals raised under the group housing conditions at the animal facility. Mice are known to form a despotic dominance hierarchy with one male being dominant and others being subordinate [44]. Social status leaves an imprint on the behavior and brain neurochemistry of mice. In this study, we obtained additional evidence that the effect of a drug may depend on the psycho-emotional state of an individual. In other words, the neurochemical background can modify the effect of a drug, sometimes yielding the opposite of the expected influence.

Moreover, some effects of a drug can be detectable in one situation (test) and undetectable in another. Apparently, the reason for this is that the predominant motivation that develops under the experimental conditions underlies many (but not all) types of behavior. Sometimes, there is a struggle between two opposite motivations (ambivalence), for example, fear and communicativeness (the test of social interactions) or anxiety and exploratory activity (the elevated plus-maze test). The balance between major motivations depends on a context, is reflected in the psycho-emotional state, and logically is influenced by the underlying neurochemical background, which may affect the biological activities of the drug. The subdivision of the defeated male mice into the subgroups—susceptible or resistant to chronic social defeat stress—has also been observed by other researchers in numerous studies [45,46,47].

In behavioral tests, chronic LiCl injections into unstressed (intact) male mice for 2 weeks were found to have anxiolytic effects in our previous study: the anxiety-like state decreased as estimated by the elevated plus-maze and social interaction tests [41]. In the present study, during preventive treatment of defeated male mice experiencing a severe anxiety-like state, the effect of LiCl correlated with the behavioral scores in various tests. Slight anxiolytic effects were observed in the partition test and decreased exploratory activities were noted in the elevated plus-maze test. Apparent anxiogenic effects of LiCl on the losers in the social interaction test may be a consequence of the frightening situation. In the group of defeated mice, LiCl had strong and varied effects: ~40% of the LiCl^+^-treated losers manifested pronounced anxiety-like behaviors in the novel environment toward the novel object and toward an unfamiliar partner under the pencil holder.

Earlier in similar experiments, LiCl has been administered preventively to male mice with repeated aggression during daily agonistic interactions accompanied by wins, as well as therapeutically to males subjected to a 20-day aggression experience with treatment in the subsequent 2-week period without agonistic interactions [41]. In that study, the preventive chronic LiCl injections into the winners were found to have well-pronounced anxiogenic effects, similar to those in the losers: LiCl further enhanced the anxiety-like state, as reported earlier for male mice, with repeated aggression in the partition test and elevated plus-maze test [41,48]. In the social interaction test, ~40% of aggressive mice demonstrated pronounced anxiety-like behavior after LiCl treatment, along with a decrease of communicativeness and exploratory activity as compared with the controls. Therefore, the anxiogenic effect of LiCl is likely to be a consequence of the stress that accompanies agonistic interactions common for aggressive and defeated mice.

During the therapeutic intervention in the no-fight period, the anxiolytic effect of LiCl became evident in the social interaction test performed on winners and was characterized by increased interest in the partner placed under the pencil holder [41]. Nevertheless, in the elevated plus-maze and partition tests, no effects of LiCl were detectable. In a study on the effects of diazepam, similar data were obtained in a slightly different experimental context: acute administration of the drug to mice with a short-term aggression experience had an anxiogenic impact, whereas in the males with a long-term experience of aggression, diazepam had an anxiolytic effect [49]. Similar results were also obtained in another work: the effects of anxiolytic chlordiazepoxide on aggressive behavior differed between animals of different social status [50].

Accordingly, the effects of chronic LiCl treatment can depend on the mode of treatment (preventive or therapeutic), on the psycho-emotional state developing during a positive or negative social experience of animals (intact, aggressive, or defeated), and on the experimental context (tests), and the effects can be anxiogenic, anxiolytic, or undetectable, as shown in our study (Table 1). 

Moreover, the response to new conditions and to anxiolytics differs among different strains of mice; this phenomenon may be explained by features of the hereditarily determined anxiety (state or trait) [51,52,53] that develops in behavioral tests. In our experiments, male mice of the C57BL/6J strain with hereditarily determined enhanced “trait” anxiety [53,54] have been used. Our data may be useful for understanding individual differences in the response to lithium in clinical practice, as reported in many studies [12,14,17].

The central role of the brain serotonergic system in the mechanisms of stress, anxiety, depression, bipolar disorder [55,56,57], and in neural plasticity [58,59,60,61,62] has been proven in numerous studies. It is widely believed that a serotonergic imbalance is a key pathophysiological mechanism of major depression. We have put forward [26] the idea that effects of LiCl treatment depend on brain serotonergic activity, which can change from day to day during the daily agonistic interactions in mice. We have previously documented an interaction between (1) a developing anxiety and depression-like state, and (2) dynamic changes in brain serotonergic activity that progress in the affected mice [26]. It was shown in that study that social defeat stress induces strong anxiety, starting from the first days of the agonistic interactions, which are accompanied by the activation of the serotonergic system. After 20 days of chronic social stress, downregulation of serotonergic genes in depressive mice is seen in midbrain raphe nuclei [35,63], which contain serotonergic neuron bodies. These data indicate links among the duration of stress, serotonergic activity, and both anxiety and depression-like states, also shown in other research on animals and humans [58,59,60,61,62]. These observations also suggest that chronic preventive administration of LiCl before and during severe stress and anxiety in animals with an experience of aggression or social defeat produces an anxiogenic effect.

In this article, we present our data on the upregulation of serotonergic genes *Tph2, Slc6a4, Htr1a*, and *Htr5b* in the midbrain raphe nuclei after preventive LiCl treatment; supposedly, these changes may lead to the activation of the serotonergic system and, as a consequence, to the development of anxiety as a side effect of LiCl. Our data are consistent with other pharmacogenomic studies which have identified candidate genes that may be sensitive to antidepressants and mood stabilizers (in particular, to lithium) including, for example, serotonergic genes *Htr2a, Htr1a, Slc6a4, Maoa,* and *Tph;* for more detail, review [33]. These results may be useful for clarifying the mechanisms of psychotropic LiCl action through the increased serotonergic gene expression and thereby serotonergic activity. Conversely, it is necessary to take into consideration that numerous other genes associated both with lithium exposure and bipolar disorder have been identified [21,22,33,64,65], and differential expression of these genes in brain tissue samples from patients and healthy controls has been investigated [66]: lithium exposure significantly affected 1108 genes, 702 of which were upregulated and 406 downregulated. Our neurogenomic data obtained in recent years by transcriptomic analysis also revealed changes in the expression of mitochondrial [67], ribosomal [68,69], monoaminergic [70,71,72,73], or autism-associated [74] genes under chronic social defeat stress. In addition, alterations in the expression of neurotrophic and transcription factors’ genes [75,76] and collagen genes [77] specific for brain regions in mice with a mixed anxiety/depression-like state have been revealed. These observations confirm that there are various mechanisms that may mediate the effects of lithium [78,79,80] at neurochemical, cellular, and genomic levels. It is becoming apparent that the research on molecular mechanisms of neuroplasticity is most promising for elucidating the pathogenesis of chronic anxiety and depression, and the efficacy of anxiolytics and antidepressants. Our behavioral approach can help to understand the effects of lithium in order to study in detail the neurogenomic mechanisms of drug action in psycho-emotional disorders.

## 5. Conclusions

The use of a pharmacological approach for screening psychotropic drugs in settings mimicking clinical conditions [36,37] allowed us to study the impact of chronic LiCl administration on anxiety-like behavior in male mice with long-term social experience of daily agonistic interactions. As shown in our study, chronic treatment with LiCl can have anxiogenic, anxiolytic, or undetectable effects, and may depend on the mode of treatment (preventive or therapeutic), on the psycho-emotional state that develops as a result of a positive or negative social experience of the animals (intact, aggressive, or defeated), and on the experimental context (tests). Our experiments suggest that preventive chronic administration of LiCl to defeated male mice under these social conditions can be accompanied by an increased expression of serotonergic genes in the midbrain raphe nuclei. Our data can clarify the individual differences in the response to lithium that are seen in clinical practice.

The limitations of this study include the following:

One should keep in mind that according to our previous neurogenomic studies, the expression of many genes in various brain regions changes under the influence of chronic agonistic interactions. Moreover, as other authors have shown, there are many genes that are sensitive to lithium. Accordingly, our results on the changes in the expression of serotonergic genes in the midbrain raphe nuclei obtained in this pharmacological study may be valid for the mixed anxiety/depression-like disorder in mice within the framework of the experimental model used. This is just the beginning of research regarding the effects of lithium treatment on the psycho-emotional state, on the one hand, and on the expression of serotonergic genes, on the other hand. Additional neurogenomic studies on the effects of lithium are needed to identify a link between the transcription of other candidate genes and anxious behaviors in mice.

Another goal is to better understand the neurogenomic mechanisms of lithium action on the expression of serotonergic and other genes, at least in the midbrain raphe nuclei, depending on the psycho-emotional state of the person, as well as the severity of the disease. It follows from this study that lithium should be given concomitantly with anxiolytics for beneficial effects; in doing so, its positive effect on the depressive state may be more pronounced. We hope that our experimental approach to studying the protective and therapeutic effects of drugs will open up new ways to more effectively treat anxiety and depressive symptoms.

## Figures and Tables

**Table 1:** Effects of chronic LiCl treatment on the anxiety-like behavior of mice with opposite social experiences.

Tests	Preventive Treatment of the Losers	Preventive Treatment of the Winners [41]	Therapeutic Treatment of the Winners [41]	Intact Males [41]
Partition	Anxiolytic effects	Anxiogenic effects	No effects	-
Plus-maze	Anxiogenic effects	Anxiogenic effects	No effects	Anxiolytic effects
Social interactions	Anxiogenic effects (40% of mice)	Anxiogenic effects (40% of mice)	Anxiolytic effects	Anxiolytic effects

## Data Availability

The statistics of the obtained data used to support the findings of this.

## Supplementary Materials

The Supplement 1 is available online at www.mdpi.com/2227-9059/9/10/1293/s1, Table S1: Primer sequences.
