# Supplementary material for: Chronic Lithium Treatment Affects Anxious Behaviors and theExpression of Serotonergic Genes in Midbrain Raphe Nuclei of Defeated Male Mice
Source: Biomedicines. 2021 Sep 22;9(10):1293. doi: 10.3390/biomedicines9101293 (PMC8533389; doi:10.3390/biomedicines9101293)
Supplement: Supplementary file 1 [file biomedicines-09-01293-s001.zip › Supplement 1.pdf]

Supplement 1.

Table S1. Primer sequences

Genes	Primer sequences		Functions
Tph2	F	5'-CACCATTGTGACCCTGAATCC-3'	Tryptophan hydroxylase 2 - rate-limiting enzyme of 5-HT synthesis
	R	5'-AAGCTCGGTGCCGTACATGAG-3'	
	P	FAM-5'-GGACGGAGGAAGAAGATCTCGA-3'-BHQ-1	
Slc6a4	F	5'- GCTGAGATGAGGAACGAAGAC-3'	Serotonin transporter protein
	R	5'- AGGAAGAAGATGATGGCAAAG-3'	
	P	FAM-5'-CCAAAGACGCGGGCCCCAGCCTC-3'-BHQ-1	
Htr1a	F	5'-TTGGAACTACTTTGGGTTATGG-3'	Serotonin 5HT1A receptors
	R	5'-ATTGTCAATTTCTTTGGTGAGTG-3'	
	P	FAM-5'-CCAATGCACAGCACCCACTTTC-3'-BHQ-1	
Htr5b	F	5'-AAAGCCGCCAAATTTCGATT-3'	Serotonin 5HT5B receptors
	R	5'-GGCTGTGAACACCATCTCAG-3'	
	P	FAM-5'-CTGCCACCACGCAGGCAAAGGAAGC-3'-BHQ-1	
B2M	F	5'-CCCCACTGAGACTGATACATAC-3'	β2-microglobulin; HLA-class I associated protein
	R	5'-GTATAGCATATTAGAAACTGGATTTG-3'	
	P	FAM-5'-GGATCGAGACATGTGATCAAGCAT-3'-BHQ-1	
